# Obituary

**Published:** 1909-12-18

**Authors:** 


### Obituary.

Dr. William Bramwel Ransom, M.A., has died at Nottingham at the age of forty-eight. Dr. Ransom, a Cambridge man, was also a B.Sc. of London, gaining a University scholarship in physiology, and the degree of M.D. in 1889. Two years later he became a member of the Royal College of Physicians, and in 1898 a Fellow. Trinity, Cambridge, and University, London, also made him Fellow, but he took the active post of physician to the General Hospital, Nottingham, and was consulting physician to the Notts Consumption Sanatorium. He was the author of several scientific papers on diverse subjects.

